# Exploring the Current Status of Risk Stratification in Hypertrophic Cardiomyopathy: From Risk Models to Promising Techniques

**DOI:** 10.3390/jcdd12030101

**Published:** 2025-03-14

**Authors:** Alexandros Kasiakogias, Christos Kaskoutis, Christos-Konstantinos Antoniou, Stavros Georgopoulos, Dimitrios Tsiachris, Petros Arsenos, Alexandrina Kouroutzoglou, Dimitrios Klettas, Charalambos Vlachopoulos, Konstantinos Tsioufis, Konstantinos Gatzoulis

**Affiliations:** First Department of Cardiology, School of Medicine, National and Kapodistrian University of Athens, Hippokration General Hospital, 11527 Athens, Greece; chris.kaskoutis@outlook.com (C.K.); ckantoniou@hotmail.gr (C.-K.A.); sta.georgopoulos@gmail.com (S.G.); dtsiachris@yahoo.com (D.T.); arspetr@otenet.gr (P.A.); alexandra211764@gmail.com (A.K.); dklettas@hotmail.com (D.K.); cvlachop@otenet.gr (C.V.); ktsioufis@gmail.com (K.T.); kgatzoul@med.uoa.gr (K.G.)

**Keywords:** hypertrophic cardiomyopathy, risk stratification, sudden cardiac death, risk models, cardiac magnetic resonance, electrophysiological study

## Abstract

Improving clinical prediction of sudden cardiac death is a crucial step in the management of patients with hypertrophic cardiomyopathy. However, finding the optimal method for risk evaluation has been challenging, given the complexity and the wide variation in clinical phenotypes. This is particularly important, as these patients are often of younger age and defibrillator implantation is associated with a low but tangible long-term risk of adverse events. A number of risk factors, including degree of hypertrophy, presence of syncope and family history of sudden cardiac death, have typically been considered to indicate a higher risk. The European risk score for prediction of sudden cardiac death is widely used; however, it may not apply well in patients with specific forms of the condition, such as those with extreme hypertrophy. Increasing evidence suggests that the presence and extent of myocardial fibrosis assessed with cardiac magnetic resonance imaging should be considered in clinical decision-making. Some research suggests that integrating electrophysiological studies into traditional risk assessment models may further optimize risk prediction and significantly improve accuracy in detecting high risk patients. Novel cardiac imaging techniques, better understanding of the genetic substrate and artificial intelligence-based algorithms may prove promising for risk refinement. The present review article provides an updated and in-depth viewpoint.

## 1. Introduction

Prevention of sudden cardiac death (SCD) is a principal step in the clinical management of patients with cardiomyopathies, and the implantation of an implantable cardioverter defibrillator (ICD) is a proven effective method for SCD prevention [1,2]. Similar to other cardiac conditions, an ICD for secondary SCD prevention is recommended following an episode of cardiac arrest. Decisions regarding ICD implantation for primary prevention are significantly more complex. There is a fine balance between implanting more ICDs to protect more people at the expense of greater rates of complications and aiming for greater specificity of at-risk individuals but with potentially more patients left exposed. At the same time, there is no consensus regarding the optimal estimated risk threshold to offer an ICD [3,4].

Hypertrophic cardiomyopathy (HCM) is the most common genetic cardiomyopathy, affecting at least one in five hundred people in the general population based on standard imaging criteria alone [5]. This condition offers a typical example of the difficulties of risk stratification decisions. The rates of SCD in HCM are low, with an annual incidence of up to 0.5–1% [6]. SCD events in patients with HCM may present at any age and irrespective of symptoms [6,7]. The advent of ICDs has reduced mortality in patients with HCM tenfold and relevant decisions are based on the assessment of predictors of increased risk [8]. Nevertheless, HCM is a largely heterogeneous condition that affects both younger and older individuals, while its increasing recognition also warrants optimization of risk stratification schemes [9].

**Figure 1:**
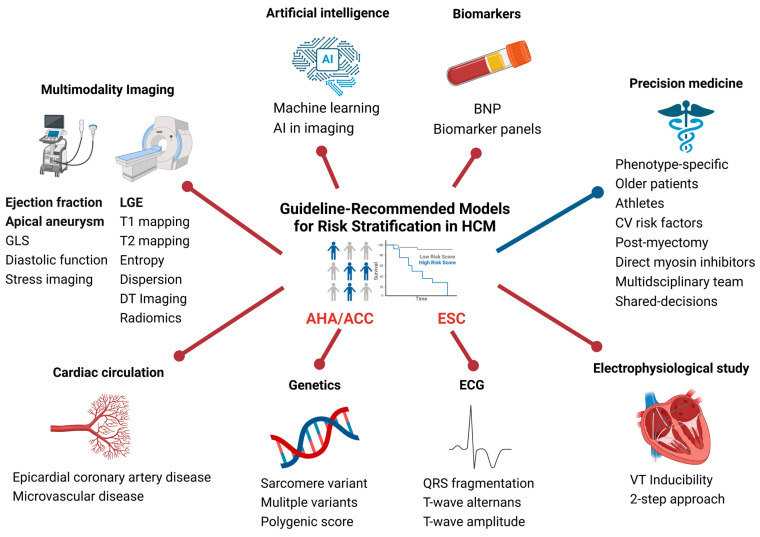
Current landscape of risk stratification methods in hypertrophic cardiomyopathy. Guidelines recommend either a major risk factor approach or an HCM SCD-Risk score-based approach. Red lines show potential contributors to risk refinement. Important information is derived from cardiac imaging (e.g., fibrosis, apical aneurysm, and systolic dysfunction). The blue line directs to considerations for an individualized approach in the context of precision medicine. For further information on each contributing factor, please consult the main text. AI, artificial intelligence; BNP, brain natriuretic peptide; GLS, global longitudinal strain; LGE, late gadolinium enhancement; HCM, hypertrophic cardiomyopathy; CV, cardiovascular; AHA, American Heart Association; ACC, American College of Cardiology; ESC, European Society of Cardiology; VT, ventricular tachycardia.

The first risk stratification criteria set for HCM were published in 2003 by an international panel with the contribution of the American College of Cardiology (ACC), American Heart Association (AHA) and European Society of Cardiology (ESC) [10]. This single-risk factor approach has been followed ever since with some variations in all AHA/ACC guidelines [2,11,12]. Since the publication of the first ESC HCM guidelines in 2014, the ESC has recommended the use of the HCM Risk-SCD tool as the main step to identify patients that would benefit from an ICD for primary prevention (Table 1) [1,3,13,14]. Over the last decade, new evidence has been emerging, with hopes of further improving the available risk prediction models. The present review aims to cover the current landscape of risk stratification for SCD in adults with HCM, from established models to new perspectives (Figure 1). The focus of the manuscript is on “sarcomeric” HCM, and phenocopies such as cardiac amyloidosis and Fabry’s disease are not considered.

## 2. Current Risk Stratification Models

The American major risk markers model follows a binary (yes/no) approach based on the presence of established risk factors for SCD in HCM [12]. The current version employs a two-step approach, initially assessing the presence of a primary tier of risk factors: massive left ventricular hypertrophy (LVH), at least one episode of likely arrhythmic syncope, a family history of SCD most likely due to HCM, LV apical aneurysm or reduced left ventricular ejection fraction (LVEF) [2]. If even one of these is present, there is a IIa indication to offer an ICD. If none of these are present, the second tier of risk factors to be reviewed includes extensive late gadolinium enhancement (LGE) or non-sustained ventricular tachycardia (NSVT). A critique of this model is that the binary approach does not allow for consideration of the different effect size of each risk factor. For instance, a maximum wall thickness (MWT) over 30 mm warrants ICD placement; however, there is a well-documented continuous association of wall thickness with risk. Additionally, the American model may be more appropriate for young and middle-aged individuals [2].

The European (ESC) model is based on the HCM Risk-SCD tool, a mathematical score that estimates the 5-year SCD risk utilizing common variables. The score has been developed based on data derived from a multicenter cohort of 3675 HCM patients, followed-up for 5.7 years. In the cohort, the following eight predictors were significantly associated with SCD at the 15% significance level: age, MWT, fractional shortening, left atrial diameter, left ventricular outflow tract (LVOT) gradient, family history of SCD, NSVT and unexplained syncope. Unexplained syncope and NSVT had the strongest association (HR of 2.53 and 2.32, respectively). The 5-year SCD risk estimates that accurately identified the highest number of SCD endpoints at 5 years, while also minimizing ICD implantation in patients without the SCD endpoint, were ≥6% for patients with two or more conventional risk factors and ≥4% for patients with one risk factor. These thresholds have been kept for the current ESC guidelines by consensus [13]. It has been shown that for every 13 ICD implantations in patients with a risk ≥6%, one patient can be saved from SCD [15].

The ESC model offers quantitative risk prediction minimizing subjective decision-making. It has been argued that the HCM Risk-SCD score does not consider the presence of extensive LGE on cardiac magnetic resonance (CMR), the presence of an apical aneurysm or LV systolic dysfunction. However, the 2023 ESC Guidelines on cardiomyopathies well offer specific mention of these newer risk arbitrators, highlighting that the available data with respect to risk prediction are more limited or less consistent and their predictive role on top of the HCM Risk-SCD score less clear [1].

There has been a long debate regarding which of the two major approaches may be superior with respect to correctly identifying most candidates that would benefit from an ICD [16,17,18]. Obviously, both strategies are effective, as documented in the significant reduction in SCD rates, despite a level of disagreement [19]. Nevertheless, balancing the risk of inappropriate shocks and other device-related complications (e.g., malfunction, thrombosis, or infection) and avoiding a tragic event is exceptionally hard. No risk model is perfect and both the European and American approaches are based on registry data, rather than randomized trials that are not feasible to conduct. In the context of HCM, even patients with no or limited symptoms and without risk factors are at a tangible risk, with an event rate of 0.6%/year [20]. At the same time, one third of patients with an ICD implanted may first receive an appropriate therapy over 10 years after device implantation [21]. The American model has been shown to present with exceptional sensitivity of over 95% in identifying at-risk patients eventually requiring ICD therapy [22,23]. Conversely, the HCM SCD-risk score presents with higher specificity, reducing the number of low-risk patients having an ICD implanted, but at the expense of significantly lower sensitivity [24]. As expected, primary prevention ICD implantation rates in US sites are twice as high as compared to non-US sites and appropriate ICD therapies are lower [19]. Several other contemporary risk model attempts to stratify patients are available, for instance with the inclusion of electrocardiographic features or by combining the major risk models; however, validation data are limited [25,26]. What needs to be highlighted is that assessment of risk and clinical decision-making, particularly for patients with intermediate risk features, should be ideally performed in the context of a multidisciplinary team meeting. The presence of cardiomyopathy specialists, cardiovascular imaging experts and clinical electrophysiologists is critical to reach consensus with respect to the management plan [27].

There are some points that are particularly relevant for clinical practice. Syncopal episodes considered to be arrhythmic are more relevant to risk stratification if they took place in the last 6 months but probably not if they took place over 5 years earlier [28]. At the same time, an arrhythmic syncope is an interim diagnosis by exclusion of other causes, performed non-invasively in most centers. The presence of NSVT is observed in 20–30% of patients with HCM and is considered in both models. However, the American guidelines place greater weight if the NSVT episodes are faster (i.e., ≥200 bpm), longer (i.e., ≥10 beats) and more frequent (i.e., ≥3 episodes in a 24 h recording) [2]. At the same time, it is less clear how episodes of NSVT documented in longer recordings (e.g., with implantable loop recorders) should be approached. In such cases, some centers calculate the HCM Risk-SCD score both with and without the NSVT. For the small number of patients who have severe LV hypertrophy (particularly if more than 35 mm), the European score underperforms as the association of risk with MWT is not linear [3]. The European score also includes the LVOT obstruction gradient, which, however, is notoriously variable and the data on its association with risk are less consistent. Of note, the presence of intracavitary obstruction should not be considered for the score calculation. Finally, the increasing availability of subcutaneous and extravascular ICDs is expected to reduce the burden of device-related complications of intravascular systems and offer excellent alternatives for young patients with an accumulating risk over a long time horizon [29,30].

## 3. Late Gadolinium Enhancement in Cardiac Magnetic Resonance

The assessment of LGE has garnered significant attention with respect to its use for risk stratification, particularly following the wider availability of CMR [1,31,32]. LGE is observed in approximately 60% of patients with HCM (Figure 1) [33]. It typically involves areas of hypertrophy and is of patchy and mid-myocardial distribution but can sometimes be transmural, as is often the case of aneurysms. The presence of LGE has been associated with greater risk of adverse outcomes, including all-cause mortality, cardiovascular mortality and SCD [34]. Of course, the high prevalence of LGE in the HCM population—which can reach over 80% in those older than 50 years—and the overall low incidence of SCD events have not allowed the LGE presence alone to be used for clinical decisions regarding protection from SCD [35].

**Figure 2:**
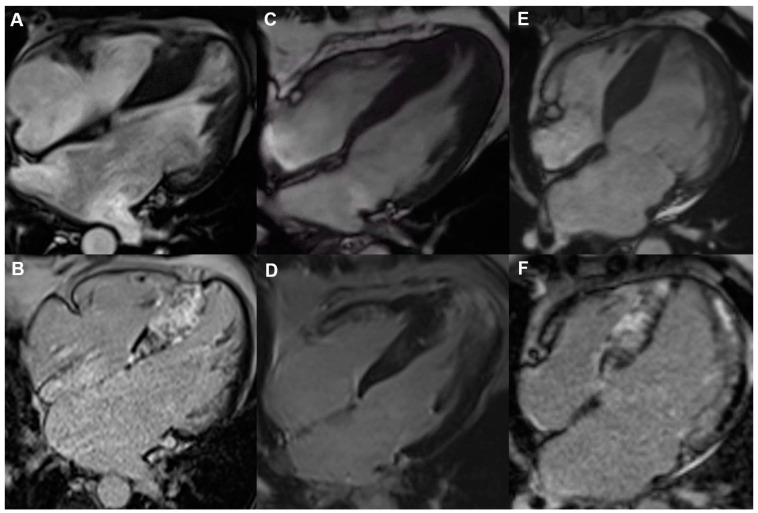
Cardiac magnetic resonance phenotypes of patients with HCM and features considered high-risk. Cine images on top and late gadolinium enhancement at the bottom. (**A**,**B**) A 53-year-old male with severe septal hypertrophy and extensive fibrosis, (**C**,**D**) a 47-year-old-female with severe apical HCM, extensive fibrosis and apical aneurysm and (**E**,**F**) a 59-year-old male with advanced HCM, severely reduced left ventricular ejection fraction and widespread fibrosis. HCM, hypertrophic cardiomyopathy.

With the increasing use of dedicated software that allows LGE quantification, the evaluation of the extent of LGE has been introduced in clinical practice as a more effective step for risk refinement (Figure 2). Choudhury et al. first described myocardial fibrosis with LGE imaging in HCM patients who are asymptomatic or mildly symptomatic and postulated the hypothesis that the mere presence of myocardial scarring is unlikely to be a risk factor for SCD but it is rather the amount of scarring that may be important [36]. A series of studies and meta-analyses have subsequently supported a stepwise increase in risk with increasing LGE severity thresholds. However, the observations have been hindered by several issues, including heterogeneity in the studied populations, different LGE quantification methods and different statistical methods used across studies, such as receiver operator characteristics analysis and quadratic spline analysis [37]. In one of the most cited studies, LGE was quantified with the 6-SD technique (signal intensity ≥6 standard deviations [SDs] above the mean of normal myocardium) in 1293 patients with HCM [38]. An LGE ≥15% was associated with an HR of 2.14 for malignant arrhythmias providing further risk discrimination over the ACC/AHA risk model utilized at the time. This cut-off was further supported by a study on 1423 patients with mostly obstructive HCM and a low–intermediate SCD risk score that documented an OR of 3.04 for a composite outcome of SCD and appropriate ICD discharge [39].

Some recent clinical data have provided further insight regarding optimal cut-offs of LGE extent for risk stratification purposes [40,41]. A retrospective analysis from China in 774 adult patients with HCM who underwent CMR examined the performance of the guideline-based models for SCD risk stratification along with the contribution of LGE [42]. Forty-six events of SCD were documented over the follow-up period of 7.4 years. LGE was assessed with the 6-SD technique. The presence of LGE ≥15% was associated with a three-fold greater risk. However, an important observation was that those patients with LGE of 5–15% were also at elevated risk and the optimal specificity and sensitivity were observed for a cut-off of about 6%. Among patients without extensive LGE, those with LGE ≥ 5% had a seven-fold greater risk. This cut-off was effective in identifying patients at increased risk that would otherwise fulfill either a class of recommendation III or II for primary prevention ICD implantation. Nevertheless, these data require further confirmation, as the clinical use of a 5% cut-off is bound to lead to a significant increase in the number of patients considered to be at elevated risk.

Along these lines, a recent, well-performed meta-analysis specifically reviewed 11 studies reporting quantitative LGE as a fraction of LV mass and examined associations with risk of SCD [43]. In total, 1550 patients with a mean age of 51 years were followed up for a median period of 5.2 years. Associations with SCD risk did not differ by differences in techniques for LGE assessment, and in six studies significant LGE was considered as 6 SDs above normal. The presence of LGE was associated with a nearly 5-fold greater risk of SCD. An LGE extent cut-off of 10% presented the best accuracy for SCD prediction (sensitivity of 0.73 and specificity of 0.67). Of note, the incidence of SCD was low and specifically at 10% in patients with LGE > 10%, while 2.4% of patients with LGE < 10% also experienced SCD.

Several other parameters have been suggested to offer prognostic information from LGE assessment. The progression of LGE may need to be considered in decision-making and this supports the need for interval CMR scan for risk stratification. In a study of 157 patients with two CMR scans over a 4.7-year interval, a high prevalence of LGE at 70% was noted at baseline and increased further to 85% in the follow-up scan, with an average LGE progression rate of 0.5 ± 1.0% per year [44]. Significant associations of the extent of LGE progression were noted with future ICD implantation, LVEF drop to ≤50% and heart failure admissions. Several factors were documented as predictors of LGE progression including a MWT of ≥20 mm, an LV mass index of ≥100 g/m^2^, an LVEF ≤60%, an LGE extent of >8% and the presence of an apical aneurysm. In another study of 105 patients with HCM who underwent two CMR scans with a 2-year interval, an increase in the percentage of patients with LGE ≥ 15% from 9 to 20% was noted [45]. An LGE progression rate (defined as LGE extent in grams over the time interval between examinations) greater than 0.07 g/month was the optimal prognostic cut-off for the study endpoint of SCD-related events.

Myocardial fibrosis detected by LGE has variable distribution and signal intensity. For instance, LGE observed at the RV insertion points has not been shown to be clearly associated with elevated risk [46,47]. Dense LGE is expected to represent replacement fibrosis, while hazy or grey-zone LGE may represent interstitial fibrosis or simply expanded areas of extracellular matrix [33]. Myocardial fibrosis that is heterogeneous and of an irregular shape could be more arrhythmogenic than homogeneous scarring. Some studies have suggested that grey-zone LGE (“mild-enhancement”) may actually predict arrhythmias more accurately than dense LGE [48]. A possible explanation is that it represents an arrhythmic substrate of viable myocytes interspersed between areas of scar tissue. For this reason, the technique of LGE dispersion mapping has been proposed, which is based on the study of 3 × 3 pixel grids over central enhanced pixels and the allocation of a dispersion score from 0 to 8 depending on how many surrounding pixels have the same signal intensity with the central pixel [49]. Among 183 patients with a low- or intermediate 5-year risk of SCD followed-up for 6 years, those with greater dispersion presented with more SCD-related events and the derived Global Dispersion Score improved risk classification when compared with LGE ≥ 15%.

## 4. Presence of a Left Ventricular Apical Aneurysm

The prevalence of an apical aneurysm is estimated at 2–5% of patients with HCM [50,51,52,53]. An apical aneurysm represents a discrete myocardial segment of the most distal LV chamber that is dyskinetic or akinetic and is typically associated with extensive to transmural scarring. The size of the aneurysm is usually measured in long-axis views in mid- to end-systole and by convention it has been considered as small when <2 cm in width, moderate-sized when 2–4 cm in width and large when >4 cm in width [50]. In the context of HCM, its presence is unrelated to coronary artery disease and typically develops in apical or mid-apical distribution of hypertrophy, with a significant proportion of patients presenting with mid-LV obstruction, or even mid-systolic doppler signal void, and an “hourglass” LV configuration [54]. There is some evidence that supports that a greater intracavitary gradient is associated with a larger aneurysm size [51]. The elevated intracavity systolic pressures and the subsequent increase in LV wall stress lead to subendocardial ischemia and eventually to myocardial scarring of the apex. However, aneurysms can also present in the absence of midventricular obstruction or cavity obliteration. Other proposed mechanisms include microvascular ischemia, increased apical oxygen-demand mismatch, genetic predisposition and myocardial bridging of the left anterior descending coronary artery [55,56].

Reports on an association of apical aneurysms with ventricular arrythmias were first published over 30 years ago [57]. In recent years, a series of small and larger observational reports have provided further insight into the role of apical aneurysms for risk stratification [50,51,52,58,59,60,61,62]. Associations of apical aneurysms have been reported for ventricular arrhythmias and SCD, heart failure and thromboembolism [58]. The area of scar tissue contiguous with the rim of the aneurysm representing the junction of viable and scarred tissue is contemplated as the substrate for ventricular arrhythmias and considered a target for successful VT ablation [63]. The relative contribution to risk of the apical aneurysm itself or the transmural scarring is still debated by some. At the same time, development of an LV aneurysm may represent disease progression to a higher-risk phenotype particularly in the presence of LV systolic impairment and documentation of NSVT [50,51].

In a landmark report, Rowin et al. retrospectively reviewed the phenotypes and associated cardiac outcomes in 93 patients of a mean age of 56 years with LV apical aneurysms who were asymptomatic or mildly symptomatic [50]. Apical type of HCM was observed in 51% of patients and mid-ventricular narrowing and obstruction in 49%. Over the mean follow-up period of 4.4 years, the SCD event rate was 4.7%/year, which was five times higher than that of patients without aneurysms. In particular, two patients experienced a sudden cardiac arrest while, out of fifty-four patients who received an ICD, eighteen experienced an aborted SCD event. Of note, most arrhythmic events represented ICD therapies for monomorphic VT, which may be unclear if they accurately represent SCD events in such patient cohorts. The size of the aneurysm ranged from 1.1 to 5.6 cm—with 57% of patients having a small aneurysm—and was not associated with outcomes; however, 71% of patients with SCD events had medium or large aneurysms.

In one of the largest retrospective cohorts to date, Lee at al. analyzed 160 patients with HCM and an apical aneurysm [51]. Their mean age was 59 years and 63% had apical HCM, while the mean aneurysm size was 1.77 cm. During a follow-up period of 6.2 years, 42% of patients had an ICD implanted and 8.8% (14 patients) experienced a SCD event. More than two thirds (71%) of those experiencing a SCD event had an aneurysm size ≥2 cm, and a linear relationship between aneurysm size and SCD risk (but also combined risk of thrombus formation and thromboembolic stroke) was observed. The authors described that an aneurysm size ≥2 cm had an approximately three-fold risk of 5-year SCD compared to <2 cm, and the same cut-off would reclassify about one third of the population to a higher risk category.

More recently, a retrospective study of 108 patients with a mean age of 57 years and apical aneurysms from three tertiary referral HCM programs reported that the incidence of significant arrythmias was associated with the presence of standard major risk factors and a larger aneurysm size [61]. Some observations agreed with previous studies. In total, 56% of patients had an ICD implanted and most arrhythmic events were VTs treated with anti-tachycardia pacing. Patients with arrhythmic events had greater aneurysm areas and volumes. Those with an echocardiographic aneurysm area >4 cm^2^ had a 5-year cumulative event rate of 35%, while patients with an aneurysm area ≤2 cm^2^ had a 5-year event rate of 6%.

Taken together, current evidence suggests that the presence of an apical aneurysm may predict arrhythmic events, although data regarding its role in refining SCD risk beyond current risk prediction models are unclear. For instance, as mentioned, whether ICD therapies for sustained monomorphic VT should be considered SCD events in the relevant studies of patients with apical aneurysms is disputed. Similarly, although some evidence directs towards larger aneurysms being associated with greater risk, the limited studies with small numbers of events precludes definitive assumptions and do not allow for a wider use of specific cut-offs. The AHA/ACC guidelines consider the sole presence of an aneurysm as a strong enough indication for an ICD, irrespective of aneurysm size or associated fibrosis. The 2023 ESC Guidelines do not include apical aneurysms in the risk stratification process but rather mention that in patients with apical aneurysms, decisions should be based on the HCM Risk- SCD score and not the presence of the aneurysm.

## 5. Left Ventricle Systolic Dysfunction

Approximately 4–8% of patients with HCM progress to a disease state with adverse LV remodeling and reduced systolic performance, typically defined as a left ventricular ejection fraction (LVEF) <50%. The underlying pathophysiological mechanism is considered to include cell necrosis, microvascular dysfunction and myocardial ischemia, and diffuse replacement fibrosis [64]. Following several small studies, recent larger registries confirm that HCM with LVSD is associated with significant morbidity and mortality, including SCD [65,66,67]. An analysis from the SHaRe registry examined 6793 HCM patients and reported that a total of 8% of patients presented with LV systolic dysfunction [66]. Patients with LV systolic dysfunction had a four times greater risk of SCD (3.9% versus 1.2%, HR: 3.9) and more frequently experienced ICD implantation (54% versus 22%), and appropriate ICD firing (25% versus 12%).

In a single-center analysis of 2447 patients with HCM, the prevalence of LV systolic dysfunction was 4.8%, with an LVEF ranging from 12 to 49% [67]. Over a mean follow-up period of 4.8 years, 48% of patients achieved clinical stability with modern heart failure therapies including cardiac resynchronization, while the remaining 52% developed refractory heart failure. Arrhythmic sudden death events were five-fold more frequent in patients with LV systolic dysfunction compared to those with preserved LVEF (2.4%/year vs. 0.5%/year). In total, 91 patients had an ICD implanted, of whom 19% experienced appropriate ICD therapy. Notably, ICD interventions were just as common in patients with LVEF of 35% to 49% versus LVEF <35%, supporting the current recommendation to offer an ICD in patients with HCM and LVEF < 50%.

Data on the risk associated with a borderline LVEF are more limited. A two-center retrospective study that categorized 1858 patients with HCM by LVEF levels—preserved (LVEF ≥ 60%), low–normal (LVEF 50–60%) and reduced (LVEF < 50%)—tracked SCD outcomes (including documented VT/fibrillation, appropriate ICD shock and aborted SCD) over a median follow-up of 4.09 years [68]. The outcome of SCD occurred in a total of 35 patients. Reduced LVEF was associated with a five-fold risk of events compared to preserved LVEF, but a low–normal LVEF did not increase the risk. Finally, in the selected patients with mild LV impairment carrying an ICD and requiring high levels of right ventricular pacing, a device upgrade to cardiac resynchronization therapy may improve systolic function, although it is less clear how this affects overall risk [69].

In the 2024 ACC/AHA Guidelines, an LVEF <50% is regarded as a major risk factor and an ICD for primary prevention should be considered in association with clinical judgment and shared decision-making. The 2023 ESC Guidelines recognize that the additional and independent prognostic value of LV systolic dysfunction on top of current risk stratification tools is less clear. Therefore, the recommendation is maintained to first estimate SCD risk using the HCM-SCD Risk tool and then to use the presence of an LVEF <50% in shared decision-making, disclosing the absence of robust data on its effect on prognosis.

## 6. Genetic Findings as Modifiers of Risk

Hypertrophic cardiomyopathy has been traditionally considered a disease of the sarcomere, and a sarcomeric gene mutation is identified in 90% of genotype-positive patients. Disease penetrance is variable but recently reported at 50% [70]. Mutations are predominantly missense or loss-of-function and causative deep intronic variants are increasingly recognized [71]. Genetic aspects that have been considered for risk stratification include the gene affected, the type of variant and the presence of multiple variants [72]. Several initial investigations that introduced the concept of genetic evaluation for risk assessment were followed by a series of observations over the following 20 years that provided contradictory results (Table 2) [73,74,75,76,77,78,79,80,81,82,83]. For many, it has been inherently impossible to rely on a specific gene mutation for risk evaluation [84]. Several factors contribute to this, including varying disease penetrance and expressivity, the large number of described variants and gene loci, variants confined to specific families, the role of other modifying genetic variation and the environmental influence. In clinical practice, attempts to include the genotype in risk stratification algorithms have not been fruitful or widely accepted, as current models integrate well-established risk factors with strong effect sizes [14].

Data regarding whether the presence of a pathogenic mutation per se may affect disease course have been conflicting as well [73,80]. Some studies support that sarcomere gene-positive patients have a greater risk of death, heart failure and ventricular arrhythmia. The Hypertrophic Cardiomyopathy Registry (HCMR) has provided insights regarding HCM phenotypes among 2755 patients from 44 sites [85]. Patients with HCM carrying a sarcomere gene variant (including variants of uncertain significance) presented with a different phenotype compared to those who were genotype-negative. Those who were genotype-positive were more likely to present with reverse curvature of the septum, more LGE and interstitial fibrosis and no significant LVOT obstruction. Conversely, those who were genotype negative were more likely to present with isolated basal septal hypertrophy, less fibrosis and, interestingly, more LVOT obstruction. Interestingly, patients with reverse curvature morphology represented most cases with LGE > 10%. Importantly, patients carrying pathogenic/likely pathogenic variants had a significantly earlier onset of total adverse events and double a risk for the ventricular arrhythmia composite compared to genotype-negative patients. These results contradict those of a recent analysis of 1468 patients from the United States and Czech Republic. In this study, patients were considered genotype positive if they were carrying a likely pathogenic or pathogenic variant and represented 21% of the cohort [86]. Over a median follow-up period of 7.8 years, 85 SCD events were documented and the cumulative incidence of SCD did not differ between genotype positive and genotype negative patients after adjusting for age and the presence of at least one major clinical risk marker.

Evidence from genome-wide association studies (GWAS) supports that variation in common genes accounts for disease expression in a significant proportion of patients with HCM and can explain disease susceptibility also in patients not carrying a rare sarcomere gene variant [87,88]. Polygenic risk scores aim to quantify the cumulative risk from common genetic variation and may become increasingly helpful for the prediction of HCM development, evaluation of relatives and, possibly, prognosis. In one of the landmark initial GWAS that involved 2780 patients with HCM and 47,486 controls, 12 genome-wide significant susceptibility loci for HCM were identified [89]. The heritability estimates of single-nucleotide polymorphisms confirmed that a substantial proportion of HCM risk was under strong polygenic influence. A polygenic score was developed, and it was shown that an increase in the score by one standard deviation unit was associated with an approximately 0.7 mm increase in MWT for both carriers of MYBPC3 truncating variants and MYH7 missense variants.

Polygenic influence is likely to affect risk prediction based on current models [1]. In a very recent study, a polygenic risk score was generated with the use of the largest available GWAS and was associated with an increased risk of HCM in a large sample from the UK Biobank. Importantly, it was possible to stratify risk of serious events among patients with HCM. Specifically, in 382 cases with HCM in the UK Biobank, a polygenic risk score in the highest quantile was associated with increased risk of death and adverse cardiovascular outcomes compared to the lowest quintile (HR of 3.88 and 3.5 respectively). Similarly, in 688 cases in the 100,000 Genome Project, a polygenic risk score in the highest quantile was associated with a six-fold higher risk of death after HCM diagnosis [90]. Nevertheless, it is still too early to fully understand the role of polygenic risk in risk stratification. Similarly to HCM risk per se, there are several parameters that need to be considered, including the interaction with rare variant carrier status and the presence of clinical risk factors such as hypertension and obesity [87,89,91,92].

## 7. Electrophysiological Testing

Unlike ischemic cardiomyopathy, where arrhythmias arise from relatively discrete scar-related reentry circuits, the arrhythmogenic substrate in HCM is diffuse and unpredictable, involving areas of fibrosis, myocyte disarray and microvascular dysfunction. The role of electrophysiological (EP) testing, and in particular programmed ventricular stimulation (PVS), for risk stratification in HCM has been assessed in several small studies over 30 years ago [93,94,95]. These early studies provided some promising results in patients considered high risk. Over the years, this option has been dismissed in guidelines based on expert opinion given the reportedly common and supposedly non-specific response of polymorphic VT with aggressive protocols, even in patients that would be otherwise considered low risk [10,13,96,97]. Nevertheless, the 2022 ESC Guidelines for the management of patients with ventricular arrhythmias mention that results have been conflicting [14]. The interventional nature of the test has further limited the use of electrophysiology testing to some expert centers. The recent ESC 2023 Guidelines on the management of cardiomyopathies and the AHA/ACC guidelines do not provide any specific recommendation on EP studies for risk stratification [1,2].

There are several intriguing concepts that support the utility of EP testing for clinical use and risk refinement in patients with HCM [41]. PVS is very safe in experienced centers and allows the direct assessment of abnormal and functional electrical circuits in the deranged myocardial architecture [98]. Syncope occurs in 20–30% of patients with HCM during their lifetime, and in about half of the cases it is regarded as unexplained. In practice, this refers to absence of vagal features or significant LVOT obstruction as well as an absence of alarming arrhythmia on Holter monitoring. An EP study allows for substrate examination for both brady- and tachycardic causes. This is particularly relevant in older ages and in the presence of multiple cardiovascular risk factors, as the underlying mechanisms may extend beyond HCM per se [92,99,100]. The combination of an EP study with an implantable loop recorder may unmask the cause of syncope and arrhythmia in selected cases [101,102]. An EP study may be helpful for the treatment of ventricular arrhythmias of an identified origin, as may sometimes be the case with VT in apical aneurysms [63]. It is also helpful in identifying the underlying mechanism of suspicious symptoms in patients with myocardial scarring following septal reduction therapies, in which both atrioventricular block and ventricular arrhythmias may be documented [103].

Along these lines, some groups have advocated shifting focus from risk-factor based models to mechanisms of arrhythmogenesis and electrophysiology-based models. A recent investigation examined five major studies on the prediction of SCD in HCM to compare the effectiveness of risk factor-based methods versus electrophysiological methods [104]. Three of the studies examined the risk factor approach [3,15,105], one was a landmark study on LGE incorporation for risk prediction [38] and the fifth study investigated paced electrogram fractionation analysis [106]. The analysis suggested that risk factor-based methods present with low predictive capability with high false positive rates (an AUC of about 0.7 and a 15–30% false positive rate). Conversely, the electrophysiological method has a higher predictive capacity with a lower false positive rate (an AUC of about 0.89 with a 20% false positive rate). The study also highlighted the misconception that good prediction involves identifying the high-risk group alone, while this is also affected by the size and mortality of the low-risk group.

One of the most contemporary studies investigating the role of EP testing was a single-center prospective study performed in 203 middle-aged patients (median age 54 years) with at least one non-invasive risk factor (family history of SCD, recent episode of unexplained syncope, NSVT, abnormal blood pressure response to exercise and MWT ≥ 30 mm) [98]. All patients underwent PVS and were followed-up for 60 months. The standardized PVS protocol consisted of up to three extrastimuli delivered at two paced cycle lengths (550 ms and 400 ms) at the right ventricular apex and right ventricular outflow tract. Extrastimuli were applied following six-beat drive trains with a 3 s interdrive pause. In total, 38.9% of patients had inducible VT. Importantly, 19 of 20 patients who met the primary endpoint of SCD or appropriate ICD discharge were inducible. The study found that PVS-guided approaches would have missed one patient with a primary endpoint, whereas AHA/ACC and ESC guidelines would have missed three and nine, respectively. A combined approach using either an ESC score ≥6% or AHA/ACC indication for an ICD with PVS inducibility resulted in a remarkable 100% sensitivity and negative predictive value. The findings lead the authors to suggest a combined approach of an ESC score ≥6% or AHA/ACC indication for ICD with PVS inducibility for clinical decision-making. In particular, a two-step-based strategy has been proposed for risk stratification. In the initial step, non-invasive risk markers are assessed, while the second step involves performing PVS to further assess the patient’s risk before considering ICD implantation.

The presence and extent of myocardial scarring has been associated with VT inducibility, and the combined characterization of myocardial fibrosis with CMR and EPS has been proposed [107]. In a study of 30 patients who underwent CMR and PVS, scar architecture examined with LGE intensity maps was correlated with features of ventricular arrhythmias that could be induced by the EP study. A gradual increase was observed in the LGE-based mass of borderzone and dense scar tissue as well as the prevalence of borderzone channels across patients who were non-inducible, inducible but without sustained VT and those with sustained VT. Additionally, a high proportion of induced ventricular arrhythmias originated as monomorphic VT at the segments were scars and borderzone channels were present [108].

## 8. Novel Approaches to Risk Stratification in Hypertrophic Cardiomyopathy

### 8.1. Sub-Phenotype-Specific Approach to Risk Stratification

Hypertrophic cardiomyopathy, similarly to other cardiomyopathies, is a largely heterogeneous disease and currently available risk models may not apply efficiently to all phenotypes met in clinical practice. Therefore, the individualization of risk prediction is key and phenotype-specific risk models could be a step forward for certain occasions. Along these lines, an apical HCM-specific risk score for the endpoint of death, appropriate ICD discharge or need for cardiac transplantation was recently developed [109]. A cohort of 462 patients with apical HCM (defined as apical wall thickness ≥15 mm) was studied, of which two thirds were asymptomatic. Interestingly, most patients (69%) had no ACC/AHA risk factors for SCD or were considered low risk based on the HCM Risk-SCD calculator (86%). The new model incorporated age, apical aneurysm, left atrial volume index, serum creatinine and right ventricular pressure. The risk score ranged between 0 and 8 points, and a higher score was associated with a higher risk. The score had good discrimination and calibration while, on the contrary, the HCM Risk-SCD calculator failed to predict the study outcome. It is, however, clear that further study is required to examine whether phenotype-specific risk models could be effective in clinical practice.

### 8.2. Multimodality Imaging

Multimodality imaging is a main component of the management of patients with HCM, with echocardiography and CMR indicated in all patients as per recent American and European guidelines [1,2,110]. The accurate measurement of MWT, left ventricular outflow tract obstruction, left atrial diameter, extent of fibrosis and myocardial contractility is important for appropriate decisions for ICD. Beyond LVEF, impaired left ventricular global longitudinal strain (LV-GLS) is an early marker of systolic dysfunction in HCM that may have predictive capacity for adverse events. A systematic review of more than 3000 patients with HCM published in 2019 identified associations of LV-GLS with ventricular arrhythmias and ICD discharge based on three studies despite significant variability in methodology [111]. In a large study of 835 patients with HCM followed-up for 6.4 years, echocardiographic LV-GLS measured with vendor-independent software was associated with a higher risk for the SCD endpoint, including appropriate ICD discharges [112]. The median LV-GLS in the study population was −15.0% and patients with LV-GLS values worse than the median had progressively higher SCD event rates. Every 1% absolute decrease in LV-GLS was associated with a 14% higher SCD risk after adjusting for the HCM Risk-SCD score, left ventricular ejection fraction and apical aneurysm. Promising results have also been published for LV-GLS based on CMR measurements [113].

Several imaging techniques based on CMR have been investigated for their prognostic ability for SCD in HCM. Parametric CMR parameters that correlate with myocardial fibrosis have been examined in the context of HCM include T1 mapping, extracellular volume, myocardial fibrosis index and T2 mapping [114]. Entropy is a radiomics measure of myocardial tissue heterogeneity and is typically derived from LGE sequences. It is independent of the signal intensity threshold, and an image with absolutely homogeneous pixels should have zero entropy. Differences in signal intensity lead to higher entropy values reflecting tissue inhomogeneity. Recent studies have reported that higher LGE entropy is associated with arrhythmic events and may improve prediction on top of LGE extent [115,116]. In a cohort of 748 patients with HCM, entropy derived from T1 mapping remained an independent predictor of the SCD endpoint after adjusting for the ESC-HCM risk score predictors and LGE greater than 15% (HR of 1.03 for each unit increase of mean entropy, 95% CI: 1.00–1.06) [117].

Diffusion tensor imaging is a CMR technique that assesses and quantifies the three-dimensional microstructure of the heart and analyzes the behavior of cardiomyocyte layers referred to as sheetlets [118,119]. In a study of 50 patients with HCM and 30 controls, fractional anisotropy, a parameter that quantifies the directionality of water diffusion along cardiac muscle fibers, was proposed as a marker of myocardial disarray. A decrease in fractional anisotropy by 0.05 increased the odds of VT of three beats or more by 2.5. Other studies have documented the role of diffuse tensor imaging-derived markers in identifying myocardial disarray and impairments in microcirculation [120,121].

### 8.3. Artificial Intelligence

Artificial intelligence and deep learning models are being investigated for both diagnostic and prognostic purposes [122,123,124]. Their utilization for risk stratification in HCM can be classified in two major groups; to increase the accuracy of measurements used in current risk stratification models and to introduce new approaches to enhance risk prediction. A CMR study in 60 patients with hypertrophic cardiomyopathy showed that an automated machine learning method trained to segment endocardial and epicardial contours outperformed international experts in the measurement of MWT [125]. This observation was based on several factors, including test–retest difference, consistency of diagnosis, minimum detectable change and standardization of measurement. Using the 30 mm MWT cutoff for ICD recommendation, the machine learning method did not reclassify any patients between test and retest scans, whereas three out of the eleven experts changed their recommendations. Machine learning methods may improve accuracy for other parameters, including the measurement of outflow tract obstruction and left atrial size [126,127]. Finally, a recent study examined the intriguing concept of electrocardiogram-based deep learning models to identify high-risk imaging features such as systolic dysfunction, massive hypertrophy, extensive fibrosis and apical aneurysm, typically best assessed with CMR [128]. The models showed good discrimination for the high-risk features and provided a paradigm to reduce the need for CMR testing in under-resourced areas.

## 9. Conclusions

Current guideline-recommended risk stratification strategies have been effective in substantially reducing rates of SCD in patients with HCM. At this stage, the next steps include a better understanding of newer parameters that may help further refine the estimated risk of each individual patient. At the same time, novel techniques will likely allow us to comprehend elusive aspects of disease development and progression. The participation of the patient in the decision-making process is very important and providing numbers of risk estimates is helpful in this respect. HCM is no longer at the stage of coming of age but at the stage of maturity. An approach that integrates both risk prediction by clinical characterization and imaging and the examination of the pathophysiological substrates of disease is welcomed.

## Figures and Tables

**Table 1:** Approach to primary prevention of SCD across clinical practice guidelines.

	ACC/ESC 2003	ACC/AHA 2011	ESC 2014	AHA/ACC 2020	ESC 2023	AHA/ACC 2024
**Risk assessment model**	Major risk factors - Family history of SCD - Unexplained syncope - NSVT - Wall thickness > 30 mm - Abnormal BP response	Major risk factors (≥1) - Family history of SCD - Unexplained syncope - NSVT - MWT > 30 mm - (Class IIa)	HCM Risk-SCD prediction score of SCD at 5 years * ≥6% (Class IIa) ≥4–6% (Class IIb) <4% (Class IIb)	Major risk factors (≥1) - Family history of SCD - Unexplained syncope (recent) - NSVT - MWT > 30 mm - Apical aneurysm - LVEF < 50% - (Class IIa)	HCM Risk-SCD prediction score of SCD at 5 years * ≥6% (Class IIa) ≥4–6% (Class IIb) <4% (Class IIb) & Risk modifiers	Major risk factors (≥1) - Family history of SCD - Unexplained syncope (recent) - NSVT - MWT > 30 mm - Apical aneurysm - LV EF < 50% - (Class IIa)
**NSVT**	Considered major risk factor	In the presence of other SCD risk modifiers (Class IIa)	Included in risk score	May be considered if no major risk factors or decision uncertain particularly if frequent, long and fast (Class IIb)	Included in risk score. There is no evidence that the frequency, duration, or rate of NSVT influences risk	May be considered if no major risk factors or decision uncertain particularly if frequent, long and fast (Class IIb)
**Abnormal** **BP response**	Considered major risk factor	In the presence of other SCD risk modifiers (Class IIa)	Not recommended	Not recommended	Not recommended	Not recommended
**LVOT obstruction gradient**	<30 mmHg compatible with lower risk	Usefulness is unclear but might be considered in selected patients (Class IIb)	Included in risk score	No mention	Included in risk score	No mention
**LV systolic function**	No mention	No mention	No mention	Major risk factor (LVEF < 50%) (Class IIa)	An LVEF <50% is a clinical risk factor to be considered in patients in the low (to intermediate) risk category (Class IIb)	Major risk factor (LVEF < 50%) (Class IIa)
**LV apical aneurysm**	No mention	May warrant consideration though evidence is limited	No recommendation due to few data	Major risk factor (Class IIa)	Decisions should not be solely based on presence of apical aneurysm	Major risk factor (Class IIa)
**Late gadolinium enhancement**	No mention	Usefulness is unclear but might be considered in selected patients (Class IIb)	No recommendation due to few data	If extensive, it may be considered if no major risk factors or decision uncertain (Class IIb)	Extensive LGE (≥15%) is a clinical risk factor to be considered in patients in the low (to intermediate) risk category (Class IIb)	If extensive, it may be considered if no major risk factors or decision uncertain (Class IIb)
**Genetic testing**	No sufficient data	Double and compound mutations (Class IIb). Otherwise, of little prognostic value	No recommendation due to few data	No mention	Not recommended	No mention
**Electrophysiology testing**	Not recommended	Not recommended	Not recommended	No mention	No mention	No mention
**Other**	Left atrium < 45 mm compatible with lower risk	SCD risk modifiers include established risk factors and emerging risk modifiers	Class IIb recommendation for ICD when risk <4% but with features of proven prognostic significance	The HCM Risk-SCD score may help patients understand the magnitude of their risk but should not be used solely for decisions.	Clinical risk factors (Risk modifiers): LVEF < 50%, LGE (≥15%)	The HCM Risk-SCD score can be useful during the shared decision-making process (Class IIa)

* Variables included in HCM Risk-SCD: MWT, LA diameter, LVOT gradient, family history of SCD, NSVT, unexplained syncope, age. BP, blood pressure; LA, left atrium; LVOT, left ventricular outflow tract; LVSD, left ventricular systolic function; MWT, maximum wall thickness, NSVT, non-sustained ventricular tachycardia; SCD, sudden cardiac death.

**Table 2:** Selected studies on clinical and prognostic information of genetic testing in HCM.

Source	Population, *n*	Registry/Cohort	Genetic Focus	Main Findings
**Watkins H et al.,** **1995 [71]**	27 families, 100 probands	Single center, USA	Troponin T, a-tropomyosin variants	Patients with variants in Troponin T presented with mild hypertrophy (maximum wall thickness 17 mm vs. 24 mm in patients with MYH7 variants, *p* < 0.001), high incidence of sudden death and a poor life expectancy of 35 years.
**Charron P et al.,** **1998 [72]**	128	Single center, France	MYBPC3 variants	Patients carrying a MYPC3 variant compared to patients carrying a MYH7 variant presented with delayed disease onset and better prognosis with no disease related deaths under the age of 40 and significantly less events of disease-related death and transplantation by 50 years of age.
**Van Driest SL et al.,** **2004 [73]**	389	Single center, USA	MYBPC3 variants	Patients with MYBPC3 variants presented with similar age at diagnosis, degree of hypertrophy, incidence of myectomy and family history of HCM or sudden death compared to patients with thick-filament HCM, thin-filament HCM or genotype-negative HCM. Patients with multiple mutations presented with the most severe disease.
**Olivotto I et al.,** **2008 [74]**	203	Two centers, Italy	Myofilament gene variants	Patients with myofilament-positive HCM showed increased risk of the combined end points of cardiovascular death, nonfatal stroke or progression to New York Heart association class III or IV compared with the patients with myofilament-negative HCM (25% vs. 7%, respectively; HR: 4.27; *p* = 0.008) and greater probability of severe left ventricular dysfunction.
**Pasquale et al.,** **2012 [75]**	92	Single center, UK	Troponin T variants	Among 20 probands and 72 relatives carrying TNNT2 variants, 22% received an ICD for primary prophylaxis and 4 died and the rate of the composite of cardiovascular death, transplantation and ICD discharge was 1.6% during a follow-up period of approximately 10 years.
**Coppini et al.,** **2014 [76]**	230	4 centers in USA and Italy	Thin filament gene variants	Patients with thin-filament variants compared to those with thick-filament HCM show milder and more atypically distributed LVH, less prevalent LVOT obstruction, higher prevalence of systolic dysfunction or restricted filling and similar rates of ventricular arrhythmias and sudden cardiac death.
**Lopes LR et al.,** **2015 [77]**	874	Single center, UK	Sarcomere gene variants	Patients with sarcomere gene variants presented with younger age, more frequent family history of HCM and sudden cardiac death, greater maximum LV wall and an increased incidence of cardiovascular death (HR: 2.81, 95% CI: 1.21–6.51, *p* = 0.012). Similar observations were reported for individual genes.
**Lee SP et al.,** **2018 [78]**	1040	SHaRe	MYH7 variants	Patients with likely pathogenic/pathogenic MYH7 variants compared to those with MYBPC3 or thin-filament variants were younger, more likely to be probands and had the highest incidence of atrial fibrillation after adjusting for age, sex, proband status and echocardiographic parameters (hazard ratio, 1.7; 95% CI, 1.1–2.6; *p* = 0.009).
**Helms A et al.,** **2020 [79]**	1316	SHaRe	Truncating MYBPC3 variants	Truncating variants accounted for 91% of MYBPC3 pathogenic variants and cause similar hypertrophy independent of location. The composite endpoint (resuscitated cardiac arrest, appropriate implantable cardioverter-defibrillator therapy, heart failure outcomes, atrial fibrillation, stroke or death) did not differ among MYBPC3 variant locations and between truncating and non-truncating variants.
**Melendo-Viu M et al.,** **2024 [80]**	188	14 centers, Spain	Truncating MYBPC3 variants	Patients with HCM carrying truncating variants presented with a low incidence of major events, most of which were heart failure. Rate of ventricular arrhythmic events was low. Neither standard risk models nor genetic factors were able to predict arrhythmic/death events. LVH showed an age-related penetrance among probands (47% at 30 years, 71% at 60 years) but the penetrance of relatives was low.

HCM, hypertrophic cardiomyopathy; LVH, left ventricular hypertrophy; LVOT, left ventricular outflow tract obstruction; SHaRe, Sarcomeric Human Cardiomyopathy Registry.

## Data Availability

No new data were generated during this study.

## Abbreviations

ACC	American College of Cardiology
AHA	American Heart Association
CMR	cardiac magnetic resonance
EP	electrophysiological
ESC	European Society of Cardiology
GWAS	genome-wide association studies
HCM	hypertrophic cardiomyopathy
ICD	implantable cardioverter defibrillator
LGE	late gadolinium enhancement
LVEF	left ventricular ejection fraction
LVH	left ventricular hypertrophy
LVMI	left ventricular mass index
LVOT	left ventricular outflow tract
LVSD	left ventricular systolic dysfunction
MWT	maximum wall thickness
NSVT	non-sustained ventricular tachycardia
PVS	programmed ventricular stimulation
SCD	sudden cardiac death
